# Development and validation of the Japanese version of the Auckland individualism and collectivism scale: relationship between individualism/collectivism and mental health

**DOI:** 10.3389/fpsyg.2024.1448461

**Published:** 2024-09-13

**Authors:** Shota Noda, Sho Okawa, Chantal Kasch, Christoph Vogelbacher, Cameron E. Lindsay, Motohiro Nishiuchi, Maaya Kobayashi, Stefan G. Hofmann

**Affiliations:** ^1^Department of Psychology, Philipps-University of Marburg, Marburg, Germany; ^2^Research Institute of Cognitive Behavior Therapy, Musashino University, Tokyo, Japan; ^3^Department of Life Sciences, The University of Tokyo, Tokyo, Japan; ^4^Research Center for Child Mental Development, Chiba University, Chiba, Japan; ^5^Graduate School of Human and Social Sciences, Musashino University, Tokyo, Japan

**Keywords:** individualism, collectivism, cross-cultural difference, mental health, J-AICS

## Abstract

**Background:**

This study developed the Japanese version of the Auckland Individualism and Collectivism Scale (J-AICS), examined its reliability and validity, and explored the associations between its factors (compete, unique, responsibility, advice, and harmony) along with variables related to mental health in the Japanese population.

**Methods:**

We recruited 476 Japanese participants from the general population. Participants completed the J-AICS along with questionnaires pertaining to culture and mental health.

**Results:**

Confirmatory factor analysis indicated the correlated five-factor model showed a good fit to the data. The Cronbach’s *α* and McDonald’s *ω* coefficients were high for the individualism, collectivism, compete, unique, and advice factors, but low for the responsibility and harmony factors. Convergent validity was supported by significant relationships between culture-related variables. A one-way analysis of variance revealed the low individualism/collectivism cluster had higher loneliness and lower satisfaction with life than the high individualism and collectivism clusters. The multiple regression analyses showed that the responsibility factor was significantly and negatively associated with mental health concerning anxiety and depressive symptoms, loneliness, and satisfaction with life. In addition, the harmony factor was significantly and positively associated with the mental health.

**Conclusion:**

These findings demonstrate sufficient validity of the J-AICS; however, reliability was insufficient for responsibility and harmony. Further, responsibility was positively associated with mental health and harmony was negatively associated with mental health.

## Introduction

Since many hallmark studies in the 1960s, the association between cultural factors and mental health have been reported across many cross-cultural studies. Most studies addressing cultural effects on mental health have focused on two dimensions of cultural factors: individualism and collectivism (e.g., Nezlek and Humphrey, 2023; Xiao, 2021). Individualism pertains to valuing personal independence, such as competition, uniqueness, and responsibility (Shulruf et al., 2007). In individualistic cultures, personal achievements and success are often the most highly rewarded and socially admired (Hofmann et al., 2010). Collectivism, on the other hand, involves valuing personal interdependence, such as advice and harmony (Shulruf et al., 2007). In collectivistic cultures, maintaining harmony within the group may be the highest priority, and individual gains may be considered less important than improvement of the broader social group (Hofmann et al., 2010). According to Hofstede et al. (2010), individualistic countries include mainly Western countries, such as the United States (U.S.), Australia, the United Kingdom, Germany, Canada, the Netherlands, and New Zealand. Collectivistic countries include mainly East Asian countries, such as Japan, Korea, and China.

Many studies have examined the associations of individualistic/collectivistic tendencies, as measured by Horizontal and Vertical Individualism and Collectivism (HVIC), with mental health. The HVIC measures vertical individualism (VI: a tendency to become distinguished and acquire status in individual competitions with others), horizontal individualism (HI: a tendency to be unique and distinct from groups without competition), vertical collectivism (VC: a tendency to perceive the self as a part of a group (mainly family) and to sacrifice their personal goals for the sake of in-group goals), and horizontal collectivism [HC: a tendency to perceive the self as part of a group and themselves and their group members as equals: (Singelis et al., 1995; Triandis and Gelfand, 1998)]. Dong et al. (2022) revealed that individualistic tendencies (the total of VI and HI) were positively associated with mental distress consisting of anxiety and depressive symptoms, while collectivistic tendencies (the total of VC and HC) were negatively associated with the mental distress among Chinese university students. Furthermore, Xiao (2021) examined the association between VI, HI, VC, HC, and mental health variables during the COVID-19 pandemic among Chinese university students. The study found that VI was positively associated with anxiety, depression, and distress, while VC was positively associated with anxiety and depression. Conversely, HC was negatively associated with anxiety and depression. Meanwhile, Nezlek and Humphrey (2023) examined the relationship between VI, HI, VC, HC, and psychological well-being variables among American university students. Their findings showed that VI was positively associated with depression and negatively associated with interpersonal well-being. Additionally, HI was positively associated with social anxiety and negatively associated with self-esteem. VC was positively associated with self-esteem and interpersonal well-being and negatively associated with depression and social anxiety, while HC was positively associated with self-esteem and interpersonal well-being and negatively associated with depression. Moreover, Germani et al. (2020) examined the association between VI, HI, VC, HC, and psychological maladjustment consisting of stress, anxiety, and difficulties among Italians. The study found that HC negatively predicted psychological maladjustment, while VI positively predicted psychological maladjustment. In contrast, there was no significant association between HI, VC, and psychological maladjustment. Humphrey et al. (2019) found that VI predicted lower mental health, including stress, depression, and anxiety among Australians, while HC predicted higher mental health. Based on these findings, the association between components of individualism/collectivism and mental health could differ from country to country.

While the HVIC is a widely used measure, it has failed to distinguish between the United States (U.S.). as an individualistic country and Japan as a collectivistic country. According to Ohashi (2004) study, there was no significant difference in VI scores between the U.S. and Japan, and scores for HI, VC, and HC were significantly higher in the U.S. than in Japan. Furthermore, Oyserman et al. (2002) reviewed previous cultural studies on individualism/collectivism and indicated that individualism/collectivism encompasses more constructs than those assumed in the HVIC, including VI, HI, VC, and HC. Specifically, they reported the following constructs for individualism: compete, uniqueness, independent, goals, private, self-know, and direct communication; and for collectivism: harmony, advice, related, belong, duty, context, hierarchy, and group oriented. Therefore, it is desirable to examine the characteristics of individualistic and collectivistic countries using the broader components of individualism/collectivism and to explore the relationship between these components and mental health.

Shulruf et al. (2007) developed the Auckland Individualism and Collectivism Scale (AICS) based on the components of individualism/collectivism identified by Oyserman et al. (2002). Shulruf et al. (2007) prepared a total of 353 items from existing scales and additional items developed by themselves. Subsequently, duplicate or similar items and items that did not fit the classification of Oyserman et al. (2002) were excluded, and finally, 66 items were extracted. Exploratory factor analysis of the 66 items revealed three factors of individualism (compete, unique, and responsibility) and three factors of collectivism (harmony, advice, and closeness). A confirmatory factor analysis (CFA) was conducted to examine the validity of the factor structure. The closeness factor did not have sufficient factor loadings. The factor was removed from the scale, and they concluded that closeness may depend on individual circumstances rather than cultural characteristics. The final version of the AICS consisted of three individualism factors (competition, uniqueness, and responsibility) and two collectivism factors (harmony and advice: 3). Previous studies adopted the two-tier hierarchical model with the compete, unique, responsibility, advice, and harmony factors as first-order factors and the individualism and collectivism as second-order factors (Shulruf et al., 2007; Shulruf et al., 2011a; Shulruf et al., 2011b). It is suggested that the psychometric properties of the scale supported its reliability, factorial validity, and robustness for comparing different populations across countries and languages (Shulruf, 2023). The AICS has been translated into 12 different languages, including Chinese, Greek, Italian, Nepalese, Portuguese, Filipino, and Turkish (Shulruf et al., 2011a; Shulruf, 2023; Bernardo et al., 2013; Kılıç and Kamaşak, 2009; Lampridis and Papastylianou, 2014; Watkins et al., 2011), making it a useful assessment tool for examining cross-cultural differences. However, to date, there has not been a Japanese-language version of the AICS. Neither has the association between cultural factors in individualism and collectivism and mental health has not been fully examined in Japan.

Therefore, this study developed a Japanese version of the AICS (J-AICS) and examined its reliability and validity. Specifically, internal consistency, factorial validity, and convergent validity were assessed. We also examined the association between cultural factors related to individualism and collectivism and variables related to mental health. By examining cultural factors as measured by the J-AICS and mental health, it is possible to identify which cultural variables are associated with mental health. This finding would contribute significantly to the understanding of culture and mental health in Japan.

## Materials and methods

### Participants

Sample size was calculated using the sample size calculator in Arifin (2023), assuming a CFI value of 0.95, mean factor loadings of 0.60, inter-factor correlation of 0.3, significant probability of 0.05, and power of 0.8. As a result, a sample of 313 participants was needed. In this study, the Directed Questions Scale (DQS; Maniaci and Rogge, 2014) was used to examine the adequacy of responses; those who did not adequately answer the items provided by two items of the DQS were excluded. Assuming that 70 percent of the total respondents responded appropriately, we recruited 500 participants from the general population via the online research company, Rakuten Insight. The participants were provided with informed consent before answering the questionnaires and received points that could be redeemed for services of Rakuten after completion of the questionnaires. Because participants were required to respond to all items, there were no missing values in this study. However, because 24 participants violated DQS items, they were excluded from the analysis. The inclusion criteria were that participants currently live in Japan and are aged 20 years or older. Overall, 476 Japanese participants (mean age = 45.59, SD = 13.55, 235 males, 241 females) were included in the study. The demographics of the participants are presented in Supplementary Table S1.

### Measures

#### J-AICS

The AICS includes 26 items and consists of five domains: compete (seven items), unique (four items), responsibility (four items), harmony (four items), and advice factors (seven items; 15). The domains of individualism are compete, unique, and responsibility factors; the domains of collectivism are harmony and advice factors. Each item is rated on a six-point scale from 1 (never or almost never) to 6 (always).

The AICS was translated into Japanese using the translation and back-translation procedure based on the consensus-based standards for the selection of health measurement instruments checklist (Mokkink et al., 2012). Initially, the first and second authors independently translated the original AICS scale from English into Japanese. The first and second authors discussed the clarity, language expression, and conceptual equivalence of the two translated AICS scales and merged them into one. Through this process, the items of the J-AICS were prepared. Thereafter, the sixth and seventh authors independently generated two back-translations based on the unified Japanese AICS scale. The first, second, sixth, and seventh authors discussed the appropriateness of the scale’s English translation as well as the original AICS scale’s contents and merged them into one. Finally, the developer of the original AICS confirmed the translation equivalence of the items. Two items were revised to reflect the original meaning of the items according to the developer’s comments, and the J-AICS was finalized. The J-AICS is presented in Supplementary material S1.

#### Culture-related variables

The following scales measuring culture-related variables were used to examine the convergent validity of the J-AICS.

#### Horizontal and vertical individualism and collectivism

To examine the convergent validity of the compete, unique, responsibility, harmony, and advice factors, the Japanese version of the HVIC developed by Tomioka (2017) was used to assess VI, HI, VC, and HC. The HVIC comprises 16 items rated on a 9-point scale ranging from 1 (never or definitely no) to 9 (always or definitely yes). The total score for each subscale ranges from 4 to 36, with a higher score indicating a greater tendency toward each cultural aspect. The HVIC scales showed low to moderate reliability in the study (VI: Cronbach’s *α* = 0.63, McDonald’s *ω* = 0.64, HI: *α* = 0.70, *ω* = 0.72, VC: *α* = 0.69, *ω* = 0.70, HC: *α* = 0.70, *ω* = 0.71).

#### Self-determination and responsibility

To examine the convergent validity of the responsibility factor, the Self-determination and Responsibility factor (SDR) in the psychological independence scale, developed by Yamada (2011), was used to assess self-determination and responsibility. The SDR comprises 9 items rated on a 5-point scale ranging from 1 (never) to 5 (always). The total score ranges from 9 to 45, with a higher score indicating a greater self-determination and responsibility. The SDR showed good reliability in this study (Cronbach’s *α* = 0.89, McDonald’s *ω* = 0.89).

#### Social support

To examine the convergent validity of the advice factor, the Japanese version of the Multidimensional Social Scale of Perceived Social Support (MSPSS), developed by Iwasa et al. (2007), was used to assess social support. The MSPSS consists of family support, significant other support, and friend support factors. The scale comprises 12 items rated on a 7-point scale ranging from 1 (very strongly disagree) to 7 (very strongly agree). The total score ranges from 12 to 84, with a higher score indicating a greater social support. The MSPSS showed good reliability in this study (Cronbach’s *α* = 0.95, McDonald’s *ω* = 0.95).

#### Cooperativeness

To examine the convergent validity of the harmony factor, the Multifaceted Cooperativeness Scale (MCS), developed by Tobari et al. (2019), was used to assess cooperativeness. The MCS consists of collaborative problem-solving, cooperation, and harmoniousness factors. The scale comprises 13 items rated on a 5-point scale ranging from 1 (not at all) to 5 (always). The total score ranges from 13 to 65, with a higher score indicating a greater cooperativeness. The MCS showed good reliability in this study (Cronbach’s *α* = 0.88, McDonald’s *ω* = 0.88).

#### Mental health-related variables

The following scales measuring mental health-related variables were used to examine the association of each factor of the J-AICS with mental health.

#### Depressive symptoms

The Japanese version of the Patient Health Questionnare-9 (PHQ-9), developed by Shimizu et al. (2013) was used to assess depressive symptoms. The PHQ-9 comprises 9 items rated on a 4-point scale ranging from 0 (not at all) to 3 (nearly every day). The total score ranges from 0 to 27, with a higher score indicating greater depressive symptoms. The PHQ-9 showed good reliability in this study (Cronbach’s *α* = 0.89, McDonald’s *ω* = 0.89).

#### Anxiety symptoms

The Japanese version of the Generalized Anxiety Disorder-7 (GAD-7), developed by Shimizu et al. (2013), was used to assess anxiety symptoms. The GAD-7 comprises 7 items rated on a 4-point scale ranging from 0 (not at all) to 3 (nearly every day). The total score ranges from 0 to 21, with a higher score indicating greater anxiety symptoms. The GAD showed good reliability in this study (Cronbach’s *α* = 0.90, McDonald’s *ω* = 0.91).

#### Loneliness

The Japanese version of the short form of the UCLA Loneliness Scale (ULS-3), developed by Igarashi (2019), was used to assess loneliness. The ULS-3 comprises 3 items rated on a 3-point scale ranging from 1 (hardly ever) to 3 (often). The total score ranges from 3 to 9, with a higher score indicating a greater loneliness. The ULS-3 showed good reliability in this study (Cronbach’s *α* = 0.84, McDonald’s *ω* = 0.85).

#### Satisfaction with life

The Japanese version of the Satisfaction With Life Scale (SWLS), developed by Sumino (Sumino, 1994), was used to assess satisfaction with life. The SWLS comprises 5 items rated on a 7-point scale ranging from 1 (strongly disagree) to 7 (strongly agree). The total score ranges from 5 to 35, with a higher score indicating a greater satisfaction with life. The SWLS showed good reliability in this study (Cronbach’s α = 0.90, McDonald’s ω = 0.91).

#### Statistical analyses

First, CFA using diagonally weighted least squares was conducted to examine the factorial validity of the J-AICS. Shulruf et al. (2007), Shulruf et al. (2011a), and Shulruf et al. (2011b) adopted the two-tier hierarchical model with the compete, unique, responsibility, advise, and harmony factors as first order factors and the individualism and collectivism as second order factors. We constructed the one-factor model, correlated two-factor model with the individualism and collectivism, correlated five-factor model with the compete, unique, responsibility, advise, and harmony factors, and two-tier hierarchical model and examined their validity by performing CFA. The comparative fit index (CFI), Tucker–Lewis index (TLI), root mean square error of approximation (RMSEA), and standardized root mean squared residual (SRMR) were computed to evaluate the model fit. The model fit is considered adequate if the CFI and TLI > 0.90 and the RMSEA and SRMR < 0.08 (Browne and Cudeck, 1993; Hu and Bentler, 1999; Van de Schoot et al., 2012; Voncken et al., 2010). The model with the highest model indices was accepted. Second, the Cronbach’s α coefficients and McDonald’s ω coefficients for individualism and collectivism dimensions and each factor of the J-AICS were calculated to assess internal consistency. Third, Pearson’s correlation coefficients between each factor of the J-AICS and the HVIC, MSPSS, SDR, and MCS were computed to examine the convergent validity of the J-AICS. Regarding the factors of the J-AICS, the compete and VI of the HVIC are similar concepts; the unique and HI of the HVIC are similar concepts; the responsibility and HI of the HVIC and self-determination and responsibility of the SDR are similar concepts; the advice and HC of the HVIC and social support of the MSPSS are similar concepts; the harmony and VC of the HVIC and cooperativeness of the MCS are similar concepts. Therefore, we assumed significant positive correlations between the above variables. Fourth, to examine the relationship between the response patterns in each factor of the J-AICS and mental health-related variables (PHQ-9, GAD-7, ULS-3, and SWLS), a cluster analysis (Ward’s method, squared-Euclidean distance) was conducted using the Z-scores of each factor of the J-AICS. Previous studies have indicated that Ward’s method is highly accurate and is the most commonly used hierarchical method in the field of health psychology (Blashfield, 1976; Clatworthy et al., 2005; Overall et al., 1993). When applying Ward’s method to measure the distance between individuals, the squared Euclidean distance is often recommended (Murase et al., 2007). We extracted the most interpretable number of clusters in the components of the individualism/collectivism response pattern based on the dendrogram for the number of clusters. Then, a one-way analysis of variance (ANOVA) was performed with each cluster as an independent variable and the mental health-related variable scores as the dependent variables. Multiple comparisons using Tukey’s method were then performed. In addition, multiple regression analysis (forced entry method) was performed with each factor of the J-AICS as independent variable and the mental health-related variables as dependent variable.

R 4.0.2 was used to employ to perform the CFA and to compete the Cronbach’s α and McDonald’s *ω* coefficients. SPSS version 28 (IBM Corp., Armonk, NY, United States) was used for correlation analysis, cluster analysis, ANOVA, and multiple regression.

### Results

#### Factorial validity

**Figure 1:**
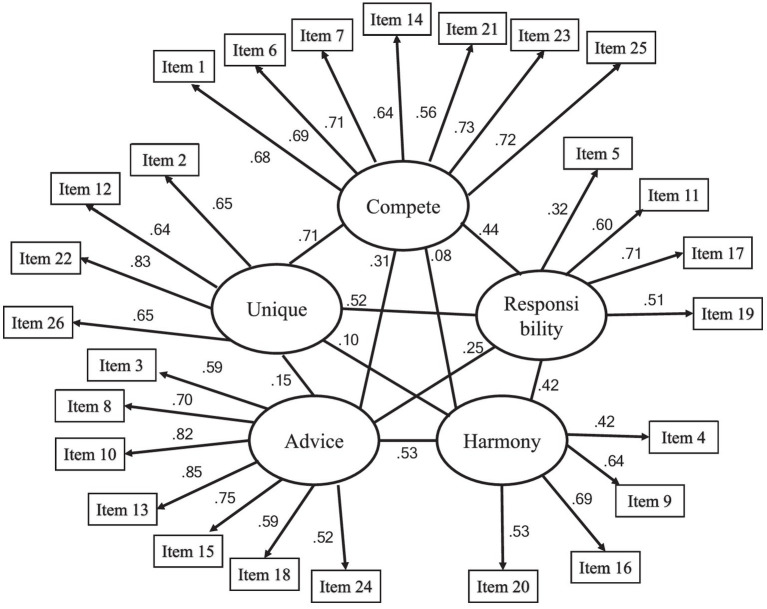
The correlated five-factor model.

Table 1 shows the fit indices for alternative factorial models. In these models, the correlated five-factor model showed a generally acceptable fit to the data and the best fit to the data {*χ*^2^ = 1027.81, df = 289, *p* < 0.01, CFI = 0.912, TLI = 0.901, RMSEA = 0.073 [90% (CI) = 0.069–0.078], and SRMR = 0.086}. All factor loadings and inter-factor correlations were significant. The correlated five-factor model is displayed in Figure 1 and the details of the other models are shown in Supplementary Figures S1–S3.

**Table 1:** Fit indices for alternative factorial models.

Model	*χ* ^2^	df	CFI	TLI	RMSEA(90%[Cl])	SRMR
One-factor model	2998.51**	299	0.677	0.649	0.138 (0.133–0.142)	0.146
Correlated two-factor model	1423.74**	298	0.865	0.853	0.089 (0.085–0.094)	0.101
Correlated five-factor model	1027.81**	289	0.912	0.901	0.073 (0.069–0.078)	0.086
Two-tier hierarchical model	1145.03**	293	0.898	0.887	0.078 (0.073–0.083)	0.090

**p < 0.01. CFI, comparative fit index; TLI, Tucker-Lewis index; RMSEA, root mean square error of approximation; 90% [CI], 90% confidence interval; SRMR, standardized root mean squared residual.

#### Internal consistency

Cronbach’s α and McDonald’s ω coefficients for the individualism/collectivism and each factor of the J-AICS were calculated. The Cronbach’s α and McDonald’s ω coefficients were high for the individualism (*α* = 0.85; *ω* = 0.86), collectivism (*α* = 0.84; *ω* = 0.85), compete (*α* = 0.85; *ω* = 0.86), unique (*α* = 0.79; *ω* = 0.79), and advice factors (*α* = 0.86; *ω* = 0.87) and low for the responsibility (*α* = 0.59; *ω* = 0.63) and harmony factors (*α* = 0.68; *ω* = 0.69).

#### Convergent validity

Results of the correlation analysis revealed a high positive correlation between the compete factor and VI (*r* = 0.70, *p* < 0.01), as well as a moderately positive correlation between the unique factor and HI (*r* = 0.53, *p* < 0.01). Furthermore, the responsibility factor exhibited weak to moderate positive correlations with the HI and SDR (*r* = 0.35, *p* < 0.01 and *r* = 0.51, *p* < 0.01), while the advice factor displayed weak to moderate positive correlations with the HC and MSPSS (*r* = 0.35, *p* < 0.01 and *r* = 0.50, *p* < 0.01). Additionally, the harmony factor exhibited a moderate positive correlation with the MCS (*r* = 0.54, *p* < 0.01). However, the harmony factor showed a very weak correlation with the VC (*r* = 0.11, *p* < 0.05). The correlation coefficients between each factor of the J-AICS and culture-related variables are displayed in Table 2 and the descriptive statistics of the participants are presented in Supplementary Table S2.

**Table 2:** Correlations between the Japanese-version of the Auckland Individualism and Collectivism Scale and culture-related variables (*N* = 476).

Scales	HVIC	HVIC	HVIC	HVIC	SDR	MSPSS	MCS
	horizontal individualism	vertical individualism	horizontal collectivism	vertical collectivism			
J-AICS							
Individualism	0.49**	0.61**	0.24**	0.24**	0.40**	0.19**	0.01
Compete	0.32**	0.70**	0.15**	0.24**	0.19**	0.11*	−0.07
Unique	0.53**	0.38**	0.19**	0.09*	0.37**	0.15**	−0.13**
Responsibility	0.35**	0.21**	0.29**	0.24**	0.51**	0.23**	0.35**
Collectivism	−0.23**	0.09	0.34**	0.25**	−0.22**	0.43**	0.43**
Advice	−0.26**	0.11*	0.35**	0.27**	−0.27**	0.50**	0.27**
Harmony	−0.07	−0.01	0.16**	0.11*	−0.03	0.12**	0.54**

**p < 0.01; *p < 0.05. J-AICS, Japanese version of the Auckland Individualism and Collectivism Scale; HVIC, Horizontal and Vertical Individualism and Collectivism; SDR, Self-determination and Responsibility factor in psychological independence; MSPSS, Multidimensional Social Scale of Perceived Social Support; MCR, Multifaceted Cooperativeness Scale.

#### Relationship between cultural factors related to individualism and collectivism and variables related to mental health

**Figure 2:**
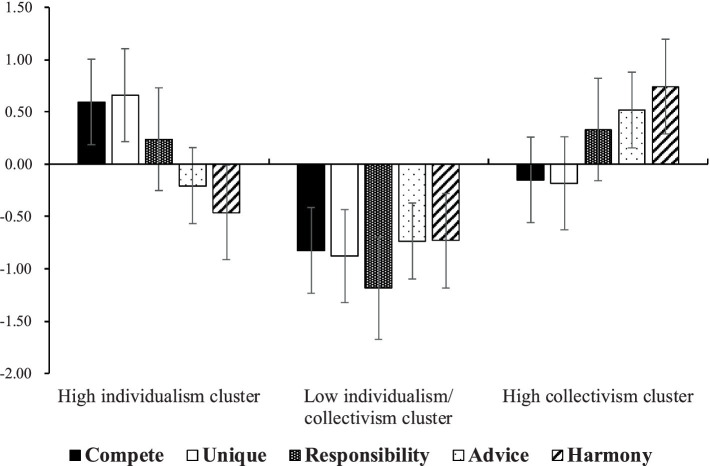
Results of the cluster analysis. The high individualism cluster has higher scores for compete, unique, and responsibility factors and lower scores for advice and harmony factors. The low individualism/collectivism cluster has lower scores for lower compete, unique, responsibility, advice, and harmony factors. The high collectivism cluster has higher scores for responsibility, advice, and harmony factors and lower scores for compete and unique factors.

The correlation coefficients between each factor of the J-AICS and variables related to mental health are presented in Supplementary Table S3. The results of the cluster analysis revealed that the five factors of the J-AICS showed three response patterns: high individualism cluster, low individualism/collectivism cluster, and high collectivism cluster (Figure 2). ANOVA showed that there were main effects of the group on the PHQ-9, USL-3, and SWLS scores [PHQ-9: *F*(2, 473) = 3.56, *p* < 0.05, η2 = 0.02, 95% CI (0.00, 0.04); USL-3: *F* (2, 473) = 4.94, *p* < 0.01, η2 = 0.02, 95% CI (0.00, 0.05); SWLS: F(2, 473) = 11.70, *p* < 0.01, η2 = 0.05, 95% CI (0.02, 0.09): Table 3]. Furthermore, multiple comparisons using Tukey’s method for the PHQ-9, USL-3, and SWLS showed significant differences. In the PHQ-9, the high collectivism cluster had significantly higher scores than the high individualism cluster (*p* < 0.05). In the USL-3, the low individualism/collectivism cluster had significantly higher scores than the high individualism and collectivism clusters (*p* < 0.05). In the SWLS, the high individualism and collectivism clusters had significantly higher scores than the low individualism/collectivism cluster (*p* < 0.01). In addition, multiple regression analysis revealed that the unique factor was significantly and positively associated with the SWLS (*β* = 0.19, *p* < 0.01). The responsibility factor exhibited significant negative associations with the PHQ-9, GAD-7, and ULS-3 (*β* = − 0.14, *p* < 0.01; *β* = − 0.12, *p* < 0.05; *β* = − 0.19, *p* < 0.01), but a positive association with the SWLS (*β* = 0.21, *p* < 0.01). The advice factor showed significant negative associations with the ULS-3 (*β* = − 0.13, *p* < 0.01), but a positive association with the SWLS (*β* = 0.20, *p* < 0.01). The harmony factor was significantly and positively associated with the PHQ-9, GAD-7, and ULS-3 (*β* = 0.24, *p* < 0.01; *β* = 0.18, *p* < 0.01; *β* = 0.12, *p* < 0.05), but negatively associated with the SWLS (*β* = − 0.16, *p* < 0.01). The results of multiple regression analysis are shown in Table 4.

**Table 3:** Descriptive statistics for each cluster and results of analysis of variance and multiple comparisons.

		(1) High individualism cluster	(2) Low individualism/ collectivism cluster	(3) High collectivism cluster	*F*-values	Multiple comparisons
		*n* = 180	*n* = 93	*n* = 203		
PHQ-9	Mean	4.88	6.37	6.22	3.56*	(1) < (3)*
	SD	4.86	5.89	5.89		
GAD-7	Mean	3.39	3.82	4.01	0.97	
	SD	4.15	4.39	4.63		
ULS-3	Mean	5.07	5.63	4.92	4.94**	(1), (3) < (2)*
	SD	1.82	2.05	1.75		
SWLS	Mean	20.80	16.65	19.51	11.70**	(2) < (1), (3)**
	SD	6.66	6.47	6.91		

**p < 0.01; *p < 0.05. PHQ-9, Patient Health Questionnare-9; GAD-7, Generalized Anxiety Disorder-7; ULS-3, short form of the UCLA Loneliness Scale; SWLS, Satisfaction With Life Scale.

**Table 4:** Results of multiple regression analysis predicting mental health.

Independent variable	Dependent variable	*R* ^2^	*β*	*t*
J-AICS	PHQ-9	*R*^2^ = 0.06**		
Compete			−0.01	−0.08
Unique			−0.04	−0.69
Responsibility			−0.14	−2.74**
Advice			−0.08	−1.53
Harmony			0.24	4.82**
J-AICS	GAD-7	*R*^2^ = 0.03**		
Compete			0.07	1.21
Unique			−0.01	−0.22
Responsibility			−0.12	−2.44*
Advice			−0.07	−1.32
Harmony			0.18	3.47**
J-AICS	ULS-3	*R*^2^ = 0.05**		
Compete			0.02	0.41
Unique			0.05	0.96
Responsibility			−0.19	−3.82**
Advice			−0.13	−2.61**
Harmony			0.12	2.39*
J-AICS	SWLS	*R*^2^ = 0.15**		
Compete			−0.01	−0.22
Unique			0.19	3.59**
Responsibility			0.21	4.44**
Advice			0.20	4.12**
Harmony			−0.16	−3.40**

**p < 0.01; *p < 0.05. *R*^2^, coefficient of determination; *β*, standardized partial regression coefficient; J-AICS, Japanese version of the Auckland Individualism and Collectivism Scale; PHQ-9, Patient Health Questionnare-9; GAD-7, Generalized Anxiety Disorder 7; ULS-3, short form of the UCLA Loneliness Scale; SWLS, Satisfaction With Life Scale.

## Discussion

The purpose of this study was to develop the J-AICS and examine its reliability and validity, as well as to determine the relationships between individualistic/collectivistic tendencies measured by the J-AICS and mental health concerning anxiety and depressive symptoms, loneliness, and satisfaction with life.

The CFA showed that the correlated five-factor model’s fit indices were generally acceptable, and the model was adopted. The Cronbach’s *α* and McDonald’s *ω* coefficients of the J-AICS for the individualism, collectivism, compete, unique, and advice factors were higher than 0.70, indicating high internal consistency. However, the Cronbach’s *α* and McDonald’s *ω* coefficients for the responsibility and harmony were low. Convergent validity was examined by assessing significant relationships between the factors of J-AICS and culture-related variables. In line with the hypothesis, the compete factor had a stronger positive correlation with VI, the unique factor had a moderate correlation with HI, the responsibility factor had weak to moderate correlations with HI and self-determination and responsibility, the advice factor had weak to moderate correlations with HC and social support, and the harmony factor had moderate correlations with cooperativeness. However, the harmony factor showed a very weak correlation with VC. This could be explained by the fact that VC measures a tendency to harmonize mainly in one’s own family, while the harmony factor of the AICS measures the tendency to harmonize with groups and others outside the family. This difference may have contributed to the low correlation. These findings demonstrate sufficient internal consistency, factorial validity, and convergent validity of the J-AICS; however, internal consistency was insufficient for the factor’s responsibility and harmony.

The original version of the AICS was developed in New Zealand, an individualistic country, and the two-tier hierarchical factor model was adopted in previous studies (Shulruf et al., 2007; Shulruf et al., 2011b). In this study, however, the two-tier hierarchical model was not supported, and the correlated five-factor model was accepted. According to Hofstede et al. (2010), Japan is classified as a collectivistic country and characterized by valuing personal interdependence. However, a previous study found that Japanese individuals are less harmony-seeking (a traditional collectivistic tendency) than those from an individualistic country, and no significant difference was found in the distinctiveness of the self (a traditional individualistic tendency) (Hashimoto and Yamagishi, 2016). Additionally, Japanese individuals have been shown to have significantly lower HI as well as HC and VC than those from the individualistic country (Ohashi, 2004). Regarding behaviors and attitudes based on collectivistic and individualistic social institutions, Japanese individuals exhibit higher rejection sensitivity in their relationships and are less self-expressive compared to those from the individualistic country (Hashimoto and Yamagishi, 2016). This is a logical tendency observed among the Japanese, who have fewer interpersonal choices, reflecting the characteristics of collectivism (Hashimoto and Yamagishi, 2016; Kusakabe et al., 2024). The J-AICS measures individual behaviors and attitudes concerning individualism and collectivism but does not control for collectivistic and individualistic social institutions. Therefore, it is speculated that the two-tier hierarchical factor model may not have been adopted. An examination of the two-tier hierarchical factor model of AICS in New Zealand, Portugal, China, Italy, and Romania suggests that there are no differences in the model between countries, except for the Chinese population (Shulruf et al., 2011a). This result may have arisen because the East Asian social institution was not considered. In the future, it is necessary to examine the factor structure of the J-AICS, considering collectivistic and individualistic social institutions.

The correlation analysis showed that competitive tendency among Japanese individuals is weakly and positively correlated with vertical collectivism. This suggests that competitive individuals in Japan may tend to place importance on group harmony, particularly within the family context. Additionally, uniqueness was found to be weakly and positively correlated with self-determination and responsibility, indicating that Japanese individuals with high uniqueness tend to take responsibility for their actions and make their own decisions to do so. Responsibility showed weak to moderate positive correlations not only with individualistic tendencies such as horizontal individualism, vertical individualism, and self-determination and responsibility, but also with collectivistic tendencies including horizontal collectivism, vertical collectivism, social support, and cooperativeness. This indicated that responsibility may be related to both collectivist and individualistic characteristics among the Japanese population. Advice was weakly to moderately and positively correlated with social support and harmony, while it was weakly and negatively correlated with horizontal individualism and self-determination and responsibility. Japanese individuals with a higher tendency to seek advice are likely to perceive greater social support and exhibit more cooperative behavior, yet may struggle with relying on themselves and making autonomous decisions. Harmony was moderately positively correlated with cooperativeness but very low positively correlated with perceived social support. This suggests that even if harmony with the group is high, Japanese individuals may not perceive that they can receive support from others. However, these findings are based on correlational analysis and do not account for extraneous variables. Future longitudinal studies that control for such variables are necessary to further validate these results.

The results of the cluster analysis showed that the Japanese sample was divided into a high individualism cluster (*n* = 180), high collectivism cluster (*n* = 203), and low individualism/collectivism cluster (*n* = 93). The high individualism cluster included those who are competitive and unique and do not seek harmony with others. The high collectivism cluster emphasizes harmony with others, seeking and conforming to others’ opinions. The low individualism/collectivism cluster indicates that all components of collectivism and individualism are low, meaning that this cluster is not adapted to Japanese culture. Notably, the high collectivism cluster also had high responsibility similar to the high individualism cluster, which differs from the AICS hypothesis that responsibility is one of the individualism dimensions (Shulruf et al., 2007; Shulruf et al., 2011a; Shulruf et al., 2011b). In other words, responsibility is generally understood as a characteristic of individualism, however, the high collectivism cluster in this study also exhibited high responsibility levels. As shown in Figure 1, there are weak to moderate positive inter-factor correlations between the responsibility, advice, and harmony factors (advice: *r* = 0.25, harmony: *r* = 0.42) in the correlated five-factor model adopted in this study, suggesting that responsibility is also related to collectivism among Japanese people. Individualistic individuals tend to view themselves as autonomous entities separate from their social context, while collectivistic individuals - such as Japanese people - tend to view themselves as connected to their social context (Markus and Kitayama, 1991). In individualistic cultures, identifying and expressing one’s internal characteristics to oneself and others is an important component of the self, while in collectivistic cultures, a fundamental aspect of one’s self-concept is the tendency to identify the needs of significant others and adjust one’s behavior accordingly (Koydemir and Essau, 2018). Individualistic individuals are idiocentric and tend to express themselves and communicate directly (Singelis, 1994; Triandis et al., 1985). Collectivistic individuals, on the other hand, are allocentric, paying attention to other people’s viewpoints and engaging in appropriate actions (Singelis, 1994; Triandis et al., 1985). Therefore, individualistic individuals have higher self-expression than Japanese individuals, while Japanese individuals have higher rejection sensitivity in their relationships with others (Hashimoto and Yamagishi, 2016). Japanese individuals tend to form relatively long-term, exclusive interpersonal relationships and groups (Kusakabe et al., 2024; Sato et al., 2014). The low relational mobility positively affects the prediction of negative evaluations by others for inferior abilities and behavior that does not conform to their social context due to the fear of being rejected by others (Kusakabe et al., 2024). Thus, it is essential for Japanese individuals to behave in order to maintain relationships with others. For example, the tendency to be modest in Japan is a strategy for adapting to the collectivistic social relationships one faces in daily life and a behavior to avoid offending others (Yamagishi et al., 2012). The responsibility factor includes consulting a supervisor and being accurate in communicating with people, which are interpreted as direct communication by Shulruf et al. (2007). In the Japanese context, however, these may be behaviors that are intended to reduce negative evaluation by others and imply behaviors or attitudes to respond to the needs of significant others, which is characteristic of collectivism. Thus, it is suggested that the responsibility factor may be related to the collectivistic tendency in Japan.

ANOVA indicated that the low individualism/collectivism cluster had higher loneliness and lower satisfaction with life than the high individualism and collectivism clusters. The low individualism/collectivism cluster, or individuals who did not conform to Japanese culture, tended to feel loneliness and had lower life satisfaction. Previous studies have also shown that those with high conflicts of cultural identity have poorer mental health, and that experiencing more culturally valued emotions (cultural fit) was linked to better general well-being (Kirchner-Häusler et al., 2023; Szabó and Ward, 2022). Previous studies have emphasized the association between cultural conflict and mental health, especially among migrant and refugee populations (e.g., Bhugra, 2004; Kirmayer et al., 2011). However, the result suggests that even among Japanese citizens, their loneliness may increase and life satisfaction may decrease when they are not adapted to their culture. Therefore, there may be a need to establish a support system for those who do not conform to the prevailing cultural norms.

The high collectivism cluster had higher depressive symptoms than the high individualism cluster, although the high collectivism cluster was similar to the high individualism cluster on the other variables. In a previous study, a collectivistic tendency was positively associated with depressive symptoms after controlling for social support (Kateri and Karademas, 2018), suggesting high harmony may be involved in increased depression. From an evolutionary psychology perspective, depression functions to avoid conflict with others, thereby enhancing harmony, and is associated with hypersensitivity to signals of social threat from others (Allen and Badcock, 2003). Japanese individuals tend to have low relational mobility, and thus have high rejection sensitivity in their relationships with others (Kusakabe et al., 2024; Sato et al., 2014). Giovazolias (2023) reported that rejection sensitivity was positively related to depressive symptoms and negatively related to perception of their relationships. Therefore, it is possible that the high collectivism cluster, because of its emphasis on harmony, tends to have higher rejection sensitivity in terms of social conformity and higher depressive symptoms. In the future, there is a need to examine the relationship between the individualism and collectivism clusters and depressive symptoms by evaluating rejection hypersensitivity.

The results of the multiple regression analysis showed that responsibility was negatively associated with anxiety, depressive symptoms, and loneliness, but positively associated with life satisfaction. Additionally, harmony was positively associated with anxiety, depressive symptoms, and loneliness, but negatively associated with life satisfaction. In the U.S., individualism is negatively related to psychological well-being, while collectivism is positively related to psychological well-being, contrary to the findings of the present study (Nezlek and Humphrey, 2023). Thus, these findings suggest that the association between individualism/collectivism and mental health may differ across countries. It should be also noted that high harmony places value on group cohesion, but for the Japanese, it may be a factor that increases their loneliness. Since it is more common for Japanese individuals not to express themselves openly (Hashimoto and Yamagishi, 2016), it is assumed that they tend to feel lonely even if they have a high degree of harmony with others. Additionally, because the Japanese have higher rejection sensitivity in their relationships as well as lower self-expression (Hashimoto and Yamagishi, 2016; Sato et al., 2014), their mental health might also be negatively affected as their tendency for harmony increases. Based on the above results, for those who do not adapt to Japanese culture, it is suggested that support that increases responsibility and decreases harmony may be effective in improving mental health. Specifically, it might be helpful to instruct them to take responsibility for their own actions in order to increase their sense of responsibility, and to encourage them to say things directly without regard to the group.

However, there are several limitations to this study that need to be considered. First, the Cronbach’s *α* and McDonald’s ω coefficients for the responsibility and harmony factors were below 0.70. A study examining the reliability of the AICS in Filipinos also reported α coefficients for the responsibility and harmony factors to be below 0.70 (responsibility factor: *α* = 0.57–0.61, harmony factor: *α* = 0.53–0.60: 16). Consistent with Zeller (Zeller, 2005), we consider an internal consistency value of 0.70 or higher as appropriate. However, it should be noted that this may decrease to 0.60 in exploratory research (Robinson et al., 1991). The number of items in the responsibility and harmony factors is relatively small, which may have contributed to the low internal consistency. The number of items in these factors should be increased to improve the internal consistency. Second, the AICS is assumed to have the two-tier hierarchical factor structure (Shulruf et al., 2007; Shulruf et al., 2011a; Shulruf et al., 2011b), but the correlated five-factor model was adopted in this study. These differences in factor structure may be related to cultural background. In the future, it is necessary to examine measurement invariance across cultures and to explore differences in cultural characteristics. Third, the model’s fit indices adopted in this study were adequate except for SRMR, which marginally exceeded the limit of 0.08. The participants in this study were Japanese individuals aged 20 and older, and differences by age and gender were not considered in the CFA. Future studies should examine the model’s fit indices while considering age and gender. Fourth, the present study used a cross-sectional design, causal relationships between the variables cannot be established based on the present results. In the future, it is necessary to examine causal relationships between the cultural factors and variables related to mental health through a longitudinal design. Fifth, this study examined culture factors and mental health in participants aged 20 years and older recruited from the general population. Although cultural tendencies may differ by generation and gender, it would be desirable to clarify cultural characteristics by generation and gender and to examine the relationship between these characteristics and mental health.

## Conclusion

The present study developed the J-AICS and demonstrated the scale’s sufficient internal consistency, factorial validity, and convergent validity. The J-AICS is expected to contribute to examining cross-cultural differences between Japan and other countries. These findings also indicate that responsibility and harmony play an important role in Japanese mental health, providing an impetus for future research comprising cross-cultural studies examining differences in mental health between Japan and other countries. Rigorous studies addressing the limitations of this study should be conducted to further examine the usefulness of the J-AICS.

## Data Availability

Detailed data are available from the corresponding author upon reasonable request.
