# Supplementary material for: Development and validation of the Japanese version of the Auckland individualism and collectivism scale: relationship between individualism/collectivism and mental health
Source: Front Psychol. 2024 Sep 13;15:1448461. doi: 10.3389/fpsyg.2024.1448461 (PMC11445612; doi:10.3389/fpsyg.2024.1448461)
Supplement: Supplementary file 1 [file Table_1.DOCX]

Table S1

The demographic data of the participants

Table S2

The descriptive statistics of the participants

Table S3

Correlations between the Japanese-version of the Auckland Individualism and Collectivism Scale and mental health-related variables

Figure S1

The one-factor model

Figure S2

The correlated two-factor model

Figure S3

The two-tier hierarchical model

Appendix S1

The Japanese version of the Auckland Individualism and Collectivism Scale

Instruction: このアンケートは，あなた自身やあなたが所属するグループについて，あなたがどのように考えたり行動したりするかを明らかにすることを目的にしています。

以下の質問を読み，それぞれの質問について，あなたが各項目に書かれているように考えたり，行動したりする頻度をお答えください。
